# ﻿Primitive new termites (Blattodea, Termitoidae) in Cretaceous amber from Myanmar

**DOI:** 10.3897/zookeys.1197.114452

**Published:** 2024-04-10

**Authors:** Yurong Jiang, Xinru Deng, Chungkun Shih, Yunyun Zhao, Dong Ren, Zhipeng Zhao

**Affiliations:** 1 Fishery Resource and Environment Research Center, Chinese Academy of Fishery Sciences, Beijing, China Capital Normal University Beijing China; 2 College of Life Sciences, Capital Normal University, Beijing, China Chinese Academy of Fishery Sciences Beijing China; 3 Department of Paleobiology, National Museum of Natural History, Smithsonian Institution, Washington, DC, USA National Museum of Natural History, Smithsonian Institution Washington, DC United States of America

**Keywords:** Fossil termites, Isoptera, Mastotermitidae, social insects, taxonomy

## Abstract

Mastotermitidae, the first-diverging extant family of termites, has only one relic extant species; however, this family had greater richness during the Mesozoic and Cenozoic eras. Fossil termites from the Cretaceous provide information on the early evolution of termites and the transition between extinct families. Herein, two new Mastotermitidae species found in upper Cretaceous (Cenomanian) Kachin amber are reported. One is a female imago described as *Angustitermesreflexus***gen. et sp. nov.** and assigned to the subfamily Mastotermitinae. The other is *Mastotermesreticulatus***sp. nov.**, which is described from an isolated forewing. With the comparison especially of the antenna and venation, these new mastotermitids further increase our knowledge of the diversity and morphology of Mastotermitidae during the Mesozoic.

## ﻿Introduction

Termites are social insects with the support of mutualistic protists or cellulose-digesting bacteria (Breznak and Brune 1994; Sanderson 1996; Sugimoto et al. 2000; Krishna et al. 2013a; Zhao et al. 2020, 2021). The enormous biomass of termites largely contributes to the global carbon cycle, while occupying an important ecological position in the biosphere (Tuma et al. 2020). The rank of termites has been controversial. Inward et al. (2007) indicated that the rank of termites should be a family, however, some termitologists proposed to continue using “Isoptera” (Lo et al. 2007), and the current compromise is to use “epifamily Termitoidae” (Eggleton et al. 2007; Bignell et al. 2011; Xiao et al. 2012; Beccaloni and Eggleton 2013; Zhao et al. 2019) or “infraorder Isoptera”.

As the first-diverging extant family in Termitoidae (Thompson et al. 2000; Legendre et al. 2015; Bucek et al. 2019; Zhao et al. 2019), Mastotermitidae shares many characters with cockroaches, for example, a large anal lobe in the hind wing and vestigial ootheca or egg pod (Nalepa and Lenz 2000; Krishna et al. 2013a). The most obvious morphological differences from other termite families are that the hind wings have large anal lobes and Sc, R, Rs, and M veins are thick and more sclerotized than CuA. Mastotermitidae was divided into two subfamilies, Mastotermitinae Desneux, 1904 and Idanotermitinae Engel, 2021, based on the presence or absence of ocelli (Jiang et al. 2021).

Besides the sole relic species, *Mastotermesdarwiniensis* Froggatt, 1897, living in northern Australia and southern New Guinea (Froggatt 1897; Nalepa and Lenz 2000; Engel et al. 2009), nine genera and 33 extinct species have been documented. Among them, 21 species were documented from compression fossils, one from Cretaceous deposits, and others from Cenozoic deposits (e.g. Wappler and Engel 2006; Krishna et al. 2013b; Vršanský and Aristov 2014; Engel et al. 2015; Bezerra et al. 2020). In addition, 12 species were reported in amber, among which seven species were reported from the Cretaceous, including five species from Kachin amber (Zhao et al. 2019; Jouault et al. 2021, 2022): *Anisotermesbourguignoni* Jouault, Engel, Huang & Nel, 2022, *Anisotermesxiai* Zhao, Eggleton & Ren, 2019, *Magnifitermeskrishnai* Jouault, Engel & Nel, 2022, *Mastotermesmonostichus* Zhao, Eggleton & Ren, 2019, *Mastotermesmyanmarensis* Jouault, 2022, *Milesitermesengeli* Jouault & Nel, 2021, one species from amber of Sagaing Region, Myanmar: *Mastotermesmyanmarensis* Jouault, 2022 (Jouault et al. 2022). One species was described from France: *Mastotermessarthensis* Schlüter, 1989 (Schlüter 1989). The other five species were reported from the Cenozoic (Krishna and Emerson 1983; Krishna and Grimaldi 1991; Nel and Bourguet 2006; Engel et al. 2007; Engel 2008).

## ﻿Materials and methods

The two specimens studied here were collected from amber mines located in the Hukawng Valley in Kachin State of northern Myanmar. Determined by U-Pb dating of Zircon, the deposit age of the amber mine was 98.79 ± 0.62 Ma which means it comes from early Cenomanian of the upper Cretaceous (Shi et al. 2012). The amber specimens involved in this study were acquired by Mr Fangyuan Xia before 2015 and donated for this study in 2016. Both specimens are deposited in the Key Laboratory of Insect Evolution and Environmental Changes, College of Life Sciences, Capital Normal University, Beijing, China (**CNUB**, curator: Dong Ren).

The amber is cut and polished. The specimens are examined, measured, and photographed with a Nikon SMZ25 microscope system. To help reduce picture distortion caused by refraction and internal cracks, the amber was placed in a properly sized crystallizing dish, with a proper amount of water, and the capture angle and area of interest was adjusted when viewing; the amber was affixed with plasticine when measurements and photographs were taken.

All images are stacked using the software Helicon Focus v. 8 for better depth of field. Simplified drawings were prepared using Adobe Illustrator CC and further modified using Adobe Photoshop CC.

## ﻿Systematic paleontology

### ﻿Family Mastotermitidae Desneux, 1904

**Subfamily Mastotermitinae Engel, 2021**

#### ﻿Genus Angustitermes

Taxon classificationAnimaliaBlattodeaMastotermitidae

Jiang, Z.Zhao & Ren gen. nov.

7ABA333A-FD6B-5710-9C32-DC61C592ED85

https://zoobank.org/FA73A948-C6EE-43D2-86E9-09485B5F7970

##### Type species.

**Figure 1.**
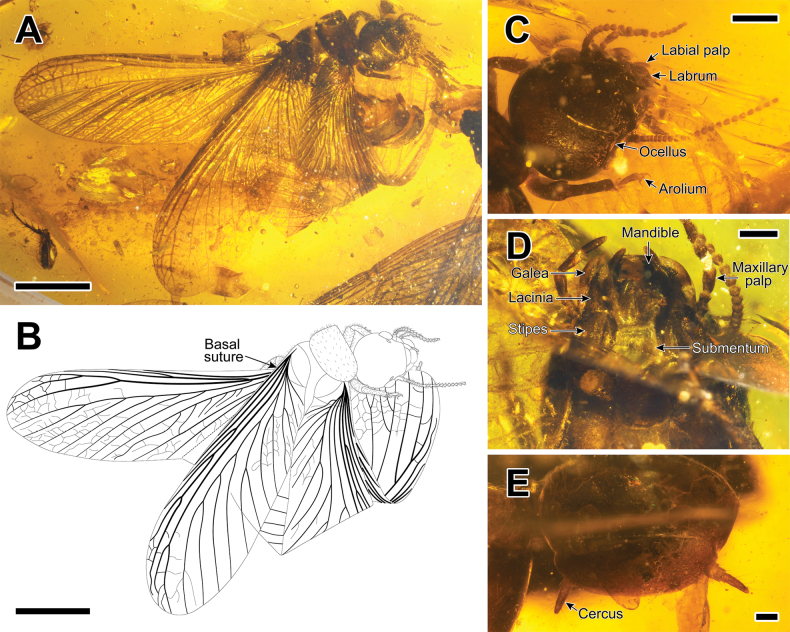
Photographs and drawing of *Angustitermesreflexus* gen. et sp. nov., holotype specimen CNU-TER-BU-2017005 (female imago) **A** habitus (dorsal view) **B** drawing of habitus (dorsal view) **C** head in dorsal view **D** head in ventral view **E** cerci in dorsal view. Scale bars: 4.0 mm (**A, B**); 1.0 mm (**C**); 0.5 mm (**D**); 0.2 mm (**E**).

**Figure 2.**
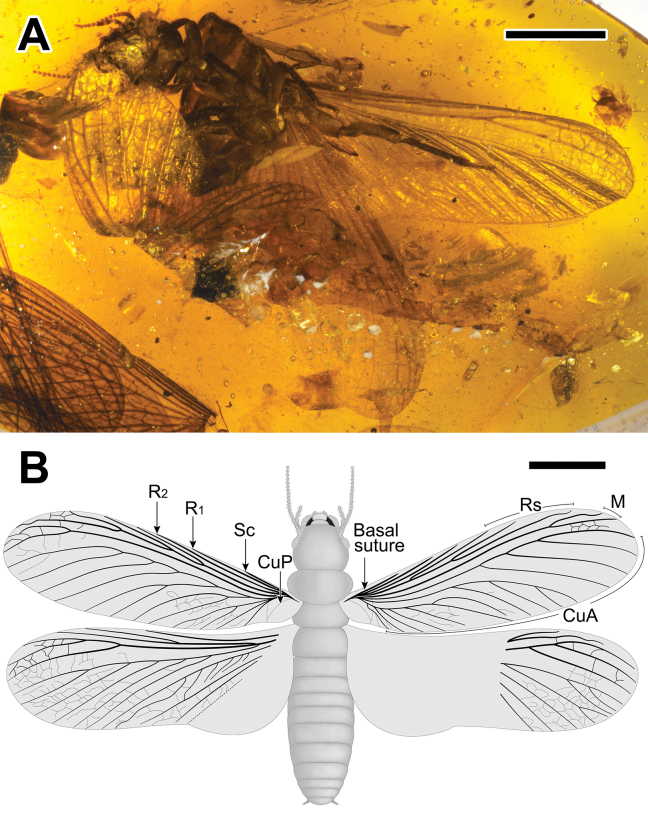
Photograph and drawing of *Angustitermesreflexus* gen. et sp. nov., holotype specimen CNU-TER-BU-2017005 (female imago) **A** habitus (ventral view) **B** partial reconstruction with wings unfolded. Scale bars: 4.0 mm (**A**); 4.0 mm (**B**).

**Figure 3.**
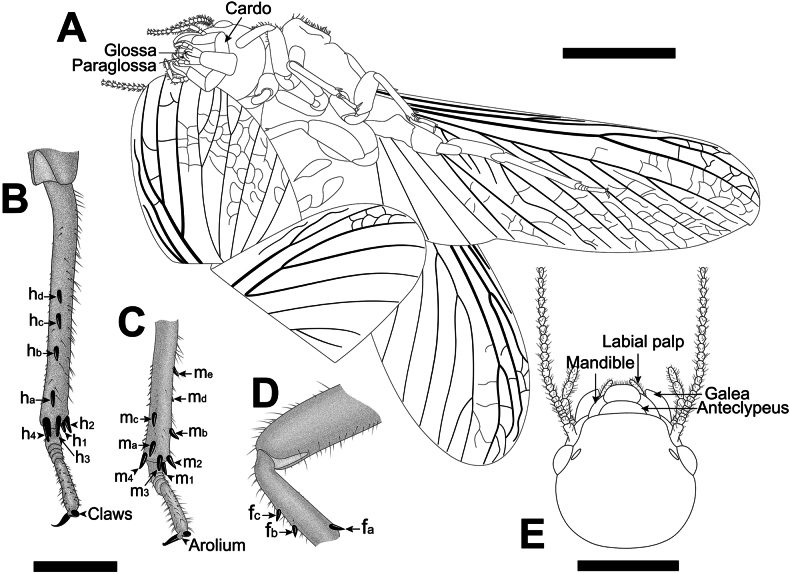
Drawings of *Angustitermesreflexus* gen. et sp. nov., holotype specimen CNU-TER-BU-2017005 (female imago) **A** drawing of habitus (ventral view) **B–D** drawings of femur, tibia, tarsi, arolium and claw of left hind leg, mid-leg and foreleg, respectively **E** drawing of head in dorsal view. Scale bars: 4.0 mm (**A**); 0.5 mm (**B, C, D**); 2.0 mm in (**E**).

*Angustitermesreflexus* Jiang, Z. Zhao & Ren, gen. et sp. nov. (Figs 1–3).

##### Etymology.

*Angusti*- is a Latin adjective, reflecting the fact that the medial field of this genus is narrow, and *termes* is the usual noun for the generic name in Termitoidae. The gender is masculine.

##### Diagnosis.

**Imago**: ocelli oval; fontanelle absent; Y-suture absent; mandibles not exceeding labrum; antenna moniliform with 22 articles; compound eyes lying in middle position on head; pronotum saddle-shaped. Wings heavily reticulated, with “cross-veins” present. **Forewing**: scale large, overlapping hind wing base, humeral suture convex; all major veins origin in scale; veins Sc, R_1_, R_2_, Rs, and M more pigmented than CuA; Sc simple; Rs with about three main branches, terminating on costal margin anterior to wing apex; radial field narrow, parallel to costal margin; M closer to Rs than CuA, lying more or less parallel to Rs as a simple vein for the greater part of its length, first branching in apical 1/5 of wing length, medial field narrow, encompassing wing apex; CuA branched, lying above the mid-longitudinal line of wing; CuP (claval suture) arched, meeting basal suture before posterior margin. **Hind wing**: basal suture not visible, large anal lobe present. **Legs**: tibial spines of all legs present; tibial spur formula 3–4–4; tarsi pentamerous; arolium present. **Abdomen**: cerci short, trimerous; abdominal styli absent.

##### Remarks.

Based on the sclerotized vein M, presence of the anal lobe, saddle-shaped pronotum, pentamerous tarsi, etc., *Angustitermes* gen. nov. is considered to belong to the Mastotermitidae and is assigned to the subfamily Mastotermitinae because of the ocelli (Fig. 1C). Besides the new genus and *Mastotermes*, there are two other genera in Mastotermitinae: *Garmitermes* Engel, Grimaldi & Krishna, 2007 and *Magnifitermes* Jouault, Engel & Nel, 2022. Termites in both *Angustitermes* gen. nov. and *Garmitermes* have a rounded head, but unlike *Angustitermes* gen. nov., *Garmitermes* has 26 antennal articles, the arolium is greatly vestigial or absent, and the tibial spur formula of *Garmitermes* is 3–5–4 (Engel et al. 2007). Both *Angustitermes* gen. nov. and *Magnifitermes* have arolium, and their tibial spur formula is the same. However, the shape of the head of *Magnifitermes* is relatively long and with about 28 antennal articles (Jouault et al. 2022). Although in different subfamilies, *Angustitermes* gen. nov. and *Anisotermes* share many similarities, such as the rounded head and the tibial spur formula. However, the M vein of *Anisotermes* is located halfway between Rs and CuA, and the first inferior branch of the M vein on the hind wings gradually fades away, ultimately terminating in the center of the hind wings of *Anisotermes* (Zhao et al. 2019; Jouault et al. 2022).

The major feature of *Angustitermes* gen. nov. is that the M vein is a simple vein for the greater part of its length, first branch branching in the apical 1/5 of wing length, and the medial field is narrow (Fig. 2B). The simple M and narrow medial field of *Angustitermes* gen. nov. is similar to *Blattotermes* Riek, 1952. However, *Angustitermes* gen. nov. has sparser veins than *Blattotermes*; the branches of R and secondary branches of CuA are fewer, and tertiary branch is absent; its forewings are much shorter, so that the difference in wing lengths between these two genera is more than 10 mm. Besides, the specimen of the new *Angustitermes* species is from the upper Cretaceous while the three *Blattotermes* species are from the Cenozoic: *Blattotermesneoxenus* Riek, 1952 and *Blattotermeswheeleri* (Collins, 1925) are from Eocene, and *Blattotermesmassiliensis* Nel, 1986 is from Oligocene (Collins 1925; Riek 1952; Nel 1986; Thorne et al. 2000; Krishna et al. 2013b).

#### ﻿ Angustitermes reflexus

Taxon classificationAnimaliaBlattodeaMastotermitidae

Jiang, Z.Zhao & Ren, gen. et sp. nov.

A050CB12-1D73-51FD-B9C2-0E4454BB75CA

https://zoobank.org/A0343631-5430-4D81-9F18-F3E5AECB4071

Figs 1 , 2 , 3

##### Type material.

***Holotype*.** Myanmar • Imago♀; Kachin State, Hukawng Valley; one imago (abdomen fragmentary); Kachin amber; CNU-TER-BU-2017005 (Figs 1–3).

##### Etymology.

The specific name, *reflexus*, means “fold”, which is in reference to the fact that the right forewing and right hindwing of the holotype are folded. The gender is masculine to match gender of the genus.

##### Locality.

The specimen was collected from the upper Cretaceous (Cenomanian) deposits of the Hukawng Valley, Kachin State, northern Myanmar.

##### Diagnosis.

As for the genus.

##### Description.

**Imago**: head length 3.38 mm, width 2.99 mm (excluding sizes of compound eyes), with sparse setae; labrum sclerotized, connecting to anteclypeus, width longer than length, anterior margin with short setae; apical tooth of mandibles not reaching the apex of labrum, first marginal tooth and apical tooth of left mandible divided in acute angle, other marginal teeth not visible; width of anteclypeus and postclypeus much longer than length; antenna with 22 articles; compound eyes hemispheric, slightly flat, diameter about 0.67 mm, lying on the middle position of the head, ommatidia diameter about 0.02 mm. Pronotum pilous, width about 3.35 mm, centraxonial length 1.97 mm, almost breaking away from the head. Wings long and broad with apex rounded, length 13.91 mm (excluding forewing scale), scale length 2.17 mm, wing width 5.02 mm; reticulated veins obviously pigmented among radial field and medial field, basal 5/8 of right hind wing hidden under right forewing. **Forewing**: scales with sparse setae, humeral margin convex, with weakly arched lobe present, basal suture curved; Sc simple, terminating on basal quarter of wing length; R with two branches in scale, terminating along anterior wing margin at half wing length; Radial field occupying about 1/8 wing area, left forewing with four branches, first branching at basal 1/3 of wing length, right forewing with three branches, first branching at basal 1/4 of wing length; M with two branches, branching at apical 1/5 of forewing; right CuA first branching in scale, occupying about 3/4 right forewing with three main branches, the branch near M vein branching at 1/2 of the wing length, the next branch branching near the middle of the wing to the base, and the branch near posterior margin branching in scale. **Hind wing**: basal suture not visible; large anal lobe present; left Rs with two branches branching from M at base; M with three branches. **Legs**: different degrees of damages to the legs, the left protarsus not preserved and the distal part of protibia not visible, the right mesotrochanter expanded and ruptured, and the large area of the left metacoxa broken; tibial spurs and spines not serrated. **Abdomen**: abdomen fragmentary, some of the detached abdominal segments can be observed, including the distal abdominal segments with a pair of cerci. Cerci trimerous; abdominal styli absent.

###### ﻿Genus *Mastotermes* Froggatt, 1897

#### ﻿ Mastotermes reticulatus

Taxon classificationAnimaliaBlattodeaMastotermitidae

Jiang, Z. Zhao & Ren sp. nov.

9D010AB3-E2F2-5E01-9C81-5E997CE1D363

https://zoobank.org/E9A4A732-D930-4B28-B22E-41507EA2C357

Fig. 4

##### Type material.

**Figure 4.**
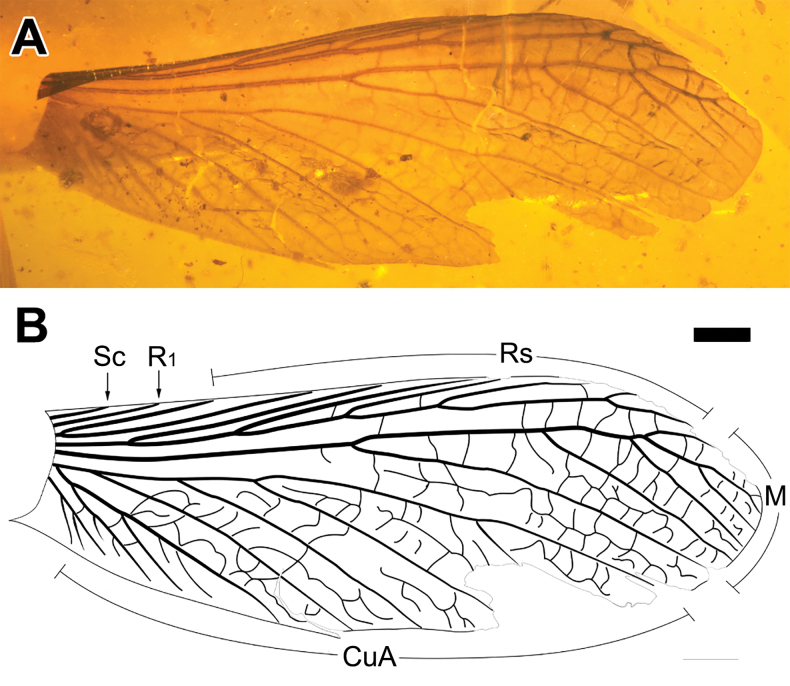
Photograph and drawing of *Mastotermesreticulatus* sp. nov., holotype specimen CNU-TER-BU-2017006 (isolated forewing) **A** photograph of forewing **B** drawing of forewing (with upturned part flattened). Scale bar: 1.0 mm (**A, B**).

***Holotype*.** Myanmar • Kachin State, Hukawng Valley; forewing (margin partly missing) only; Kachin amber; CNU-TER-BU-2017006 (Fig. 4).

##### Etymology.

Latin, *reticulatus*, meaning “reticulate”, which is in reference to the well-developed and clearly recognizable reticulated veins of holotype.

##### Locality.

The specimen was collected from the upper Cretaceous (Cenomanian) deposits of the Hukawng Valley, Kachin State, northern Myanmar.

##### Diagnosis.

**Forewing**: wing length (excluding forewing scale) about three times as long as wide; forewing scale large; heavily reticulated, irregular bilayer structure in M and CuA, cross veins between Rs and M obvious; Sc, R, Rs, and M thickened, strongly sclerotized and obviously pigmented; Sc and R_1_ simple and short; Rs with seven or eight branches, with secondary branches, running parallel to costal margin; M with six branches, first branch of M arising at forewing mid-length, secondary branches present, medial field encompassing wing apex, angle of branches and main vein gradually increasing.

##### Description.

**Forewing**: forewing length (excluding forewing scale) 13.2 mm, width about 4.7 mm; suture length about 2.3 mm, strongly arched, making a sharp angle with posterior margin; distal costal margin slightly missing, posterior margin partially missing because of abrasion of the amber; base of costal margin rolled up, making the Sc and R poorly visible; Sc length 1.0 mm; R_1_ length 2.0 mm, Rs with eight branches; M slightly closer to Rs than to CuA, encompassing wing apex, six branches visible, angle of branches and main vein gradually increasing, so that branches terminate closely on margin, first branch of M arising at forewing mid-length, second branch arising at about distal third of forewing length, and base of third branch close to fourth branch with secondary branch.

##### Remarks.

The forewing of *Mastotermesreticulatus* sp. nov. is similar to that of *Mastotermesmonostichus* Zhao, Eggleton & Ren, 2019, but there are some differences; the Sc, R, and Rs of *Mastotermesreticulatus* sp. nov. are closer, and there are fewer secondary branches, which makes the Rs veins parallel to the costal margin, instead of gradually narrowing as in *M.monostichus*. Compared with *M.monostichus*, the M vein of *M.reticulatus* sp. nov. is even more irregular and with fewer branches (Fig. 4). These different characteristics indicate that they are not the same species.

## ﻿Discussion

The number of antennal articles in *Angustitermesreflexus* gen. et sp. nov. is only 22 (Fig. 2B), which is fewer than in most mastotermitids, especially the range of 29–32 of the extant *Mastotermesdarwiniensis* (Krishna et al. 2013a), even though the antennae of many fossil termites are incompletely preserved. Another mastotermitid, *Mastotermesminutus* Nel & Bourguet, 2006 (Nel and Bourguet 2006), which was described from Early Eocene amber of Oise (France), has the fewest antennal articles, with only 20. Other mastotermitids that have been reported with complete antennae have more than 24 antennal articles. In addition to the antennae and the synapomorphies of mastotermitids, there are many similarities between *Mastotermesminutus* and *Angustitermesreflexus* gen. et sp. nov.: an ocellus is present, which indicates that they belong to the same subfamily; M of the hind wing branches from Rs, close to the wing scale; meso- and metatibiae have at least three lateral spines; tibial spurs are 3–4–4; and an arolium is present. However, these species also have some differences: the M vein of *Angustitermesreflexus* gen. et sp. nov. is closer to Rs than Cu (Fig. 2B), while the M vein of *Mastotermesminutus* is closer to Cu than to Rs. In terms of geological time, the number of antennal articles within Mastotermitidae seems to change from many (plesiomorphic state) to few and then back to many. Further phylogenetic analysis with more fossil mastotermitids may clarify the specific trend of the evolution of the number of antennal articles in Mastotermitidae.

The sclerotized vein M of the forewings in *Angustitermesreflexus* gen. et sp. nov. are relatively simple, with only two branches and the medial fields, located in the anterior third, compressed. Concurrently, the distal-most branches of CuA, which end at the wing apex, are also thick and sclerotized, similar to M, and stronger than the other branches of CuA (Figs 2, 3A). This is opposite in the wings of *Mastotermesreticulatus* sp. nov., *Anisotermesxiai*, and *Mastotermesmonostichus*, which have multibranched M in the forewing, with the basal-most branches gradually tapering and unsclerotized. Mastotermitids share a synapomorphy: the Sc, R, Rs, and M veins are thicker and much sclerotized than CuA (Engel et al. 2009; Zhao et al. 2019). The synapomorphy tends to indicate the overall degree of sclerotization present in each vein, and some branches with different locations may show different degrees of thickness and sclerotization.

## Supplementary Material

XML Treatment for Angustitermes

XML Treatment for Angustitermes reflexus

XML Treatment for Mastotermes reticulatus

## ﻿Additional information

### Author ORCIDs

Yurong Jiang https://orcid.org/0000-0002-7249-1377

Chungkun Shih https://orcid.org/0000-0002-3434-2477

Dong Ren https://orcid.org/0000-0001-8660-0901

Zhipeng Zhao https://orcid.org/0000-0002-6665-8943

### Data availability

All of the data that support the findings of this study are available in the main text.
